# Minimally Invasive Distal Pancreatectomy Is Associated with Decreased Postoperative Neutrophil to Lymphocyte Ratio

**DOI:** 10.1089/pancan.2019.0020

**Published:** 2020-05-12

**Authors:** Richard Zheng, Olivia Wang, Emma Bradley, Harish Lavu, Jordan R. Winter, Ernest L. Rosato, Francesco Palazzo, Charles J. Yeo, Adam C. Berger

**Affiliations:** ^1^ Department of Surgery and the Jefferson Pancreas, Biliary, and Related Cancer Center, Thomas Jefferson University Hospital, Sidney Kimmel Medical College, Philadelphia University and Thomas Jefferson University, Philadelphia, Pennsylvania, USA.; ^2^ Department of Surgery, Lankenau Medical Center, Wynnewood, Pennsylvania, USA.; ^3^ Department of Surgery, University Hospitals Cleveland Medical Center, Case Western Reserve University School of Medicine, Cleveland, Ohio, USA.; ^4^ Department of Surgery, Rutgers Cancer Institute of New Jersey, New Brunswick, New Jersey, USA.

**Keywords:** minimally invasive, distal pancreatectomy, neutrophil to lymphocyte ratio, inflammation

## Abstract

**Purpose:** The neutrophil-to-lymphocyte ratio (NLR) is a marker of inflammation that has been investigated as a prognostic factor in many diseases. We hypothesized that NLR would be lower in patients undergoing minimally invasive distal pancreatectomy (MIDP).

**Methods:** Using a prospective database, we identified patients who underwent open or minimally invasive (laparoscopic/robotic) distal pancreatectomy and splenectomy from 2006 to 2018. Patients were grouped according to their type of surgery and matched by age, gender, and benign or malignant pathology. The NLR was calculated from a complete blood count with differential on the second postoperative day. Statistical calculations were performed in Stata (v13.0).

**Results:** A total of 106 patients were included, with 53 MIDP and 53 open cases. MIDP was associated with a significantly lower postoperative NLR than open surgery (13.3 vs. 17.2, *p* = 0.01). NLR did not vary significantly between patients who developed complications and those who did not (15.4 vs. 15.3, *p* = 0.95). Patients undergoing MIDP had decreased length of postoperative hospital stay (4 days vs. 5 days, *p* = 0.003). Multivariable linear regression failed to find a significant decrease in NLR with the use of laparoscopy (*p* = 0.14) when accounting for age, body mass index, surgical blood loss, pathology, and operative time as covariates.

**Conclusion:** The NLR is significantly decreased when performing MIDP versus open distal pancreatectomy, but correlation with clinical outcomes has yet to be proven.

## Introduction

Excessive activation of the body's own systemic inflammatory response has been negatively implicated in various disease processes. An elevated systemic response, as measured by circulating levels of well-known biomarkers such as C-reactive protein (CRP) or erythrocyte sedimentation rate, is associated with increased postoperative complications and decreased cancer-specific survival.^1,2^ Laparoscopic and robotic surgery have been associated with a decreased inflammatory response compared with laparotomy according to certain inflammatory markers such as IL-6, TNF-α, and CRP.^3^ Similarly, patients undergoing laparotomy appear to have impaired cell-mediated immune function compared with their laparoscopic counterparts.^4^ These tests, however, are not commonly ordered or routinely assessed in the postoperative period.

The neutrophil-to-lymphocyte ratio (NLR) is another such marker that has recently been investigated as a prognostic factor in cancer and other diseases. Unlike other similar measures of inflammation such as serum CRP or the Glasgow prognostic score (CRP:albumin),^5^ the NLR is easily calculated from a complete blood count (CBC) with a manual differential, which is routine laboratory testing that almost all surgeons obtain during an inpatient stay. Moreover, there is now increasing evidence that elevated NLR values are associated with worse cancer-specific outcomes^6–7^; with regard to pancreatic cancer, elevated preoperative NLR values are inversely associated with overall and disease-free survival after surgical resection.^8–14^

Postoperative NLR, by comparison, has been much less studied; some studies that have measured postoperative NLR immediately after major surgery have found elevated postoperative NLR to be a significant predictor of risk, associated with increased cancer-specific recurrence and higher complication rates after gastrectomy, bariatric surgery, esophagectomy, and colorectal surgery.^15–20^ However, there is significant variability in NLR levels after major surgery among these studies, and little acceptance of what is normal. As a result, there is no accepted “standard” for postoperative NLR as there are for other well-established measures of inflammation, and there have been no studies comparing postoperative NLR between different approaches to the same standard surgical technique, that is, distal pancreatectomy. We have chosen to study the postoperative NLR to address this gap in knowledge, and hope to find a practical use for this accessible but poorly defined metric.

We assume that the postoperative NLR, unlike the preoperative NLR, will be representative of the inflammatory processes that are set in motion by surgery. As others have shown using animal models and different biomarkers,^21,22^ we suspected that minimally invasive approaches would cause less inflammation and lead to decreased morbidity. Therefore, we hypothesized that patients who underwent minimally invasive distal pancreatectomy (MIDP) would have a decreased NLR compared with those undergoing the same open procedure (distal pancreatectomy) during a contemporary time frame. We also secondarily investigated the association between NLR and postoperative complications and outcomes.

## Materials and Methods

This study was approved by the institutional review board at Thomas Jefferson University Hospital.

### Patient selection and NLR calculation

**FIG. 1.**
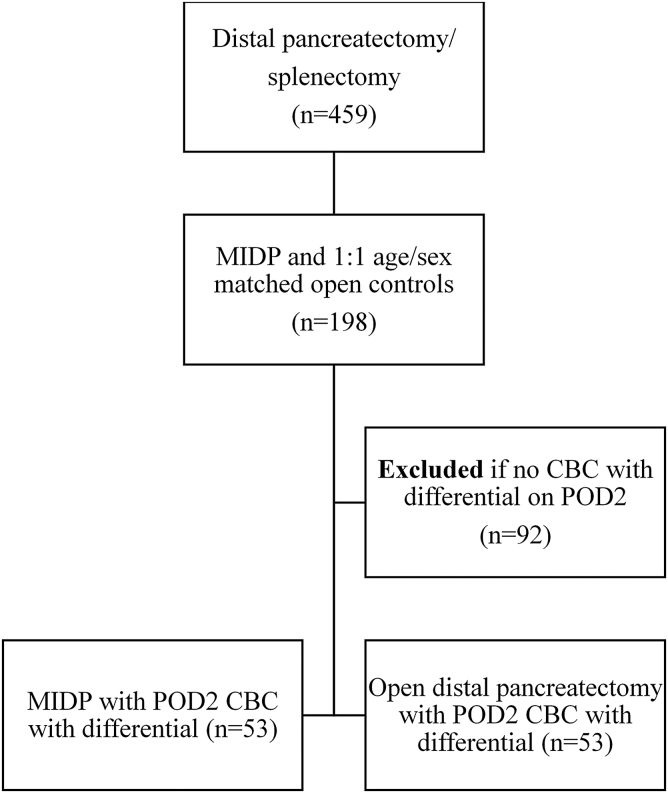
Flowchart determining case eligibility. Flowchart demonstrating selection criteria for cases of distal pancreatectomy. CBC, complete blood count; MIDP, minimally invasive distal pancreatectomy.

A retrospective review was performed on a prospectively maintained institutional pancreatic surgery database. Patients were identified who underwent laparoscopic or robotic distal pancreatectomy (MIDP) and splenectomy from 2006 to 2018. We determined that the largest number of these patients had a CBC with differential on the second postoperative day (POD2). Therefore, patients without blood counts drawn on POD2 were excluded to preserve consistency in comparing NLR between groups. The remaining patients were matched by age, gender, and pathology with those who underwent open distal pancreatectomy during the same time frame and also had appropriate CBC results on POD2 (Fig. 1).

Although we would have ideally included equal proportions of patients with ductal adenocarcinoma in both groups, until recently, MIDPs have not been as commonly performed for ductal adenocarcinoma, which limited our ability to match equal numbers of patients with pancreatic ductal adenocarcinoma (PDA). Instead, matching based upon pathology was performed to ensure an even distribution of benign and malignant cases between both groups. For the purposes of this study, PDA, acinar cell carcinoma, pancreatic neuroendocrine tumors (PNETs), and mucinous cystic neoplasms (MCNs) with invasive components were considered malignant. All other pathologies were deemed to be benign.

Eight different surgeons were responsible for performing open distal pancreatectomy; five of these surgeons performed MIDP. Postoperative NLRs were calculated from the CBCs that were drawn on POD2 (in the first 24 h after surgery, our surgeons obtain only hemoglobin levels without an accompanying full CBC). The first neutrophil and lymphocyte counts from that day were used, regardless of white blood cell count. Demographic statistics recorded included age, race, gender, smoking and diabetes status, and final pathology of the resected specimen.

### Operative technique

All open distal pancreatectomies were performed in a similar manner with an accompanying splenectomy. A generous midline incision was used to gain access to the peritoneal cavity. Laparoscopic and robotic distal pancreatectomy and splenectomy was performed with a mixture of trocar sizes that varied between surgeons at our institution, but most commonly included three to four 5 mm trocars and two to three 12 mm trocars. Operative time and estimated surgical blood loss were included in multivariable analysis.

### Postoperative outcomes

Postoperative outcomes measured include complications, blood loss, postoperative transfusions (at any point during their initial hospital stay), duration of surgery, length of stay, recurrence, and mortality among patients with invasive cancers. Complications were graded according to the Clavien–Dindo classification system. Postoperative pancreatic fistulae (POPF) were defined according to the International Study Group in Pancreatic Surgery (ISGPS) as intraperitoneal drain amylase levels greater than three times the upper limit of normal.

### Statistical analysis

Student's *t*-tests and Fisher's exact tests were used to compare categorical variables with a significance level of 0.05. Univariate and multivariable regression models were used to determine the relationship between NLR and continuous patient-related or operative characteristics. All statistical analysis was carried out with Stata version 13.0 (StataCorp, 2013, College Station, TX).

## Results

### Demographics

The study group consisted of 106 age-, gender-, and pathology-matched (with respect to malignant or benign disease) patients who underwent distal pancreatectomy and splenectomy during the study period (Table 1). Of these, 53 underwent open resection and the other 53 had an MIDP (30 robotic and 23 laparoscopic cases). The average age of all patients was 64 years. There were 30 men and 23 women in each cohort. The mean body mass index (BMI) was 28 in both groups. There were 19 (35.8%) patients undergoing open pancreatectomy who were active or former smokers, as compared with 24 (45.2%) in the MIDP group. There were 16 patients with diabetes in the open group (30.2%) and 12 in the MIDP group (22.6%).

**Table 1.** Demographics of Patients Undergoing Distal Pancreatectomy and Splenectomy

Variable	Open	MIDP	Total	p
Age (years)				
Mean	62.9	64.5	63.7	0.46
Median	64	65	64	
Median year of surgery performed	2012	2013	2013	0.98
Median length of follow-up (months)	75	70	72.5	0.91
Gender				
Male	30	30	60	0.93
Female	23	23	46	
Body mass index (kg/m^2^)				
Mean	28.0	29.2	28.1	0.42
Median	28	28	28	
Race				
African American	5	3	8	0.18
Other	1	4	5	
White	47	46	93	
Diabetes				
Type II	16	12	30	0.38
Smoking				
Never smoker	33	29	62	0.65
Former smoker	17	21	38	
Active smoker	2	3	5	
Pathology				
Malignant disease	25	25	50	1.00
Primary invasive carcinoma^a^	16	8	24	
Neuroendocrine tumor	8	17	25	
Metastatic lesion	1	0	1	
Benign disease	28	28	56	
IPMN	7	13	20	
Benign cystic lesion^b^	6	7	13	
Other benign lesion^c^	2	5	7	
MCN	3	2	5	
Pancreatitis	9	0	9	
Solid pseudopapillary	1	1	2	

^a^ PDA, adenosquamous carcinoma, IPMN or MCN with invasive component, spindle cell.

^b^ IOPN, pseudocyst, cystadenoma/simple cyst.

^c^ Nesidioblastosis, fibrosis, Pan-IN.

IOPN, intraductal oncocytic papillary neoplasm; IPMN, intraductal papillary mucinous neoplasm; MCN, mucinous cystic neoplasm; MIDP, minimally invasive distal pancreatectomy; PDA, pancreatic ductal adenocarcinoma.

There were 25 cases of malignant disease in each cohort, but there were more instances of invasive carcinoma among open cases (*n* = 16, 30.2%) than in MIDP cases (*n* = 8, 15.1%). PNET was the most common pathology, with 8 (15.1%) in the open group and 17 (32.1%) in the MIDP group; as discussed previously, for the purposes of this study, we considered them malignant due to potential for recurrence and metastasis. Intraductal papillary mucinous neoplasms (IPMNs) and cystic lesions made up the remaining bulk of benign resected pathologies (no IPMNs with components of invasive carcinoma were included in this sample).

### Outcomes

The mean operative time in the open cohort was 4.9 h versus 5.1 h in the minimally invasive cohort (*p* = 0.65) (Table 2). Complication rates were similar between groups, with 15 (28.3%) in the open group and 19 (35.8%) in the minimally invasive group. There were nine patients with major complications (Clavien–Dindo class III or greater) in all, with five in the open group and four in the MIDP group (*p* = 0.30). These major complications included pancreatic fistulae or abscess requiring drain placement (*n* = 5), intraperitoneal bleeding requiring reoperation (*n* = 2), and early small bowel obstruction requiring reoperation (*n* = 2). In total, there were 22 POPF, with 8 in the open group (15.1%) and 14 (26.4%) in the laparoscopic cohort (*p* = 0.239).

**Table 2.** Outcomes and Complications After Distal Pancreatectomy

Variable	Open (n = 53)	% Total	MIDP (n = 53)	% Total	p
WBC^a^					
Mean	18.3		19.2		0.33
Neutrophil %					
Mean	64.8		52.4		**0.05**
Lymphocyte %					
Mean	4.4		5.5		0.22
NLR					
Mean	17.2		13.3		**0.01**
Median	15.6		12.5		
EBL (mL)					
Mean	587		153		**0.002**
OR time (h)					
Mean	4.9		5.1		0.65
Transfusion					
Total	5		1		0.09
Complications					
No complication	38	35.8	34	32.1	0.30
Clavien–Dindo I	8	7.5	7	6.6	
Clavien–Dindo II	2	1.9	8	7.5	
Clavien–Dindo III	4	3.8	4	3.8	
Clavien–Dindo IV	1	0.9	0	0.0	
POPF					
Total	8	7.5	14	13.2	0.23
Postoperative hospital length of stay					
Mean (days)	5.6		4.9		**0.003**
Median (days)	5		4		
Recurrence^b^					
Total	8	7.5	4	3.8	0.37

Bolded values represent significant values at a level of *p* < 0.05.

^a^ Collected on postoperative day 2.

^b^ With regard to malignancy only.

EBL, estimated blood loss; NLR, neutrophil-to-lymphocyte ratio; OR, operating room; POPF, postoperative pancreatic fistula; WBC, white blood cell count.

The median postoperative hospital length of stay was shorter in the MIDP group versus the open group (4 days vs. 5 days, *p* = 0.003). The median lengths of postoperative follow-up for the open and MIDP groups were 75 and 70 months, respectively.

Among patients undergoing surgery for invasive carcinoma, there were eight recurrences in patients with invasive carcinoma in the open group (*n* = 8/16, 32%) and four (*n* = 4/8, 50%) in the minimally invasive group (*p* = 0.37). Median postoperative follow-up times for the entire cohort for open and MIDP patients with malignant disease (*n* = 50) were 58 and 48.5 months, respectively (*p* = 0.54). The mean NLR was significantly higher in those with invasive cancers that recurred (20.0 vs. 14, *p* = 0.03).

### Comparing NLR between groups

**FIG. 2.**
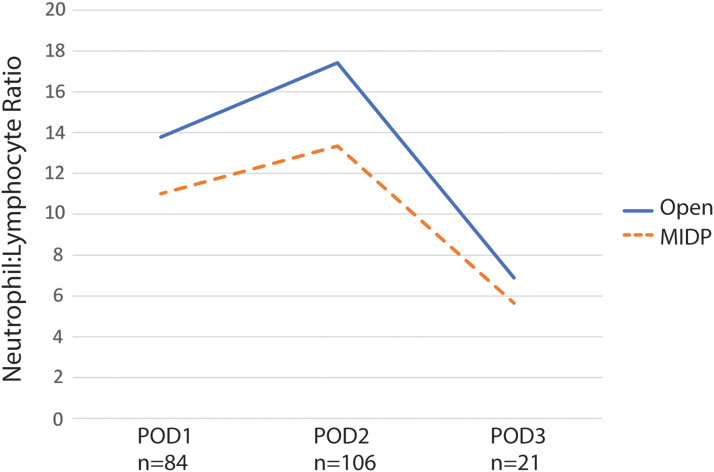
Neutrophil:lymphocyte ratio after distal pancreatectomy by POD. POD, postoperative day.

The mean POD2 NLR in the open cohort was significantly higher than the NLR of the MIDP cohort (17.2 vs. 13.3, *p* = 0.01). Among subjects who had a POD1 differential (*n* = 84/106, 79.2%), the mean POD1 NLR in the open cohort was 13.8 versus 11.0 in the MIDP cohort (*p* = 0.30). Among the subset of subjects who also had a POD3 differential (*n* = 11/106, 10.4%), the mean POD3 NLR in the open cohort was 6.9 versus 5.6 (*p* = 0.23; Fig. 2). There was no significant difference in mean NLR between patients undergoing laparoscopic and robotic surgery (*p* = 0.09). In addition, there was no association between mean NLR and the occurrence of complications (15.4 vs. 15.3, *p* = 0.95) or being transfused with red blood cells (15.2 vs. 16.8, *p* = 0.64).

The NLR did not differ significantly in the open surgery group between those with and without complications (17.7 vs. 17.1, *p* = 0.83); this is also true of those who underwent MIDP with versus without complications (13.5 vs. 13.2, *p* = 0.87). We found no difference in the NLR between those who developed a POPF compared with those who did not develop a POPF (15.6 vs. 14.2, *p* = 0.46). When controlling for pathology within the MIDP cohort, patients with true invasive cancers (*n* = 8) had a similar NLR to those who did not (14.1 vs. 13.2, *p* = 0.73). Within the open cohort, patients with invasive cancers (*n* = 17) had an NLR of 19.9 versus 16.0 in patients who did not have an invasive cancer (*p* = 0.13).

Similarly, the NLR did not vary significantly between patients with and without PNET undergoing open surgery (17.3 vs. 16.6, *p* = 0.83), but the mean NLR was lower in patients with PNET undergoing MIDP compared with patients without MIDP (10.9 vs. 14.6, *p* = 0.05). Finally, there was also no significant difference in mean NLR between patients operated on for invasive cancers or those with benign disease (15.2 vs. 15.4, *p* = 0.87; Table 3). When calculated for specific pathologies, mean NLR was highest among patients with PDA (18.2 ± 8.7), followed by MCN (16.8 ± 9.8), IPMN (16.4 ± 9.7), benign cystic disease (12.8 ± 5.7), and PNET (12.6 ± 7).

**Table 3.** Associations of Operative Variables with Mean Neutrophil-to-Lymphocyte Ratio

Variable	Mean NLR	N	95% CI	p
Open surgery	17.2	53	14.9–19.6	
MIDP (total)	13.3	53	11.5–15.2	**0.01**
No complication (total)	15.3	72	13.2–17.3	
Complication (total)	15.4	34	13.1–17.6	0.95
No complication (open)	17.1	38	14.1–20.0	
Complication (open)	17.7	15	13.4–21.9	0.83
No complication (MIDP)	13.2	34	10.6–15.9	
Complication (MIDP)	13.5	19	11.3–15.8	0.87
Nonsmoker	15.2	101	13.6–16.8	
Active smoker	16.9	5	7.5–26.2	0.66
No transfusion	15.2	100	13.6–16.8	
Any transfusion	16.8	6	7.6–25.9	0.64
No POPF	15.6	84	13.8–17.4	
POPF	14.2	22	11.3–17.1	0.46
Nondiabetic	15.2	78	13.3–17.0	0.80
Diabetic	15.6	28	12.8–18.4	
Benign	56	15.2	13.1–17.2	0.87
Malignant	50	15.4	13.1–17.8	
Recurrence^a^	38	14.0	11.6–16.3	**0.03**
No recurrence^a^	12	20.0	13.8–26.2	

Bolded values represent significant values at a level of *p* < 0.05.

^a^ Only calculated for patients with invasive carcinoma.

### Multivariable regression analysis

Regression analysis of age, BMI, operative time, operative blood loss, and pathology failed to find any significant association with regard to postoperative NLR at the level of *p* = 0.05 (Table 4).

**Table 4.** Multivariate Regression for Predictors of Neutrophil-to-Lymphocyte Ratio

Variable	Regression coefficient	p	95% CI
MIDP	−6.1	0.14	−14.3 to 2.1
Body mass index (kg/m^2^)	0.20	0.60	−0.5 to 0.8
Operative length (min)	0.24	0.85	−2.4 to 2.9
Estimated blood loss (mL)	−0.001	0.68	−0.004 to 0.003
Preoperative WBC	1.5	0.15	−0.6 to 3.6
Age (years)	−0.03	0.84	−0.32 to 0.3
Malignant disease	−6.4	0.09	−13.9 to 1.1

## Discussion

Our study finds that, in the setting of distal pancreatectomy, laparoscopic surgical techniques lead to lower values of postoperative NLR independently of other factors. We think it is reasonable to assume that this occurs due to the decreased systemic inflammatory response associated with minimally invasive surgery compared with traditional open distal pancreatectomy. The NLR also did not vary significantly with demographic factors such as age or BMI, or with other comorbidities such as diabetes. Although neither the NLR nor minimally invasive techniques appear to reliably predict outcomes, we did not anticipate finding such differences due to the imbalances in invasive carcinoma between the groups. The focus of our study was to demonstrate that NLR is decreased with minimally invasive surgery—a simple statement that, until now, had not been validated using a procedure that is still commonly performed by both open and minimally invasive means.

The NLR is a measure that is low-cost, easily used, and quickly interpretable, but has been underutilized in the postoperative setting; we demonstrate that the NLR correlates with surgical technique and establish benchmarks for its use as a marker of the inflammatory response after pancreatic surgery. Although we fail to find a significant association with postoperative NLR and recurrence in our study, chronic inflammation has been strongly linked to pancreatic carcinogenesis,^23^ it is possible that future studies with a larger scope may find that the postoperative NLR is a useful predictor of disease-free survival and oncological outcome.

Similar to other studies before us, we were not able to demonstrate a significant relationship between choice of surgical approach and the occurrence of complications. Twelve nonrandomized studies have similarly failed to show a significant difference in complication rates between laparoscopic or open distal pancreatectomy.^15^ In particular, subjects who developed POPF—a common and often problematic complication after distal pancreatectomy—did not have a significantly different NLR from those who did not. The reasons for this are unclear, but this higher rate of POPF in the laparoscopic group may offset any decrease in complication rates that would otherwise accompany a decreased systemic inflammatory response. Complications after distal pancreatectomy appear to be multifactorial; other patient- and disease-related factors are known to influence complication rates independent of the degree of inflammation caused by distal pancreatectomy alone.^16,17^ Certain subpopulations that have a higher baseline risk of major complications, such as the frail and elderly, derive a significant morbidity benefit from MIDP^18^; further study could find that similar subpopulations are more prone to suffering complications related to an overactive inflammatory response.

Although the acute inflammatory reaction that occurs in the first few days after surgery makes these values difficult to interpret, we specifically wanted to study how the peak inflammatory response—as influenced by surgical technique—could be a determinant of outcomes. Use of the postoperative NLR as a prognostic tool for other surgeries will require some standardization with regard to the timing of measurement. Prior studies of postoperative NLR have largely measured NLR several weeks after surgery, when most of the immediate inflammatory response has abated. In contrast, we chose the NLR on POD2 as our primary end-point; surgeons at our institution most often obtain their first postoperative CBC with differential on POD2, and our NLR data are most robust on that day.

The pitfall of using postoperative NLR in the acute postoperative setting is that it varies greatly between procedures, with the upper limit of normal ranging from 2 to 50 in prior studies depending on the procedure. Although the NLR values in our study may not be generalizable to all procedures, the postoperative NLR values seen here may be applicable to all distal pancreatectomies and may additionally shed light on the reduction in NLR that is to be expected with the minimally invasive version of any particular procedure. To our knowledge, ours is the first study to specifically compare postoperative NLR between open and minimally invasive versions of the same surgery, performed contemporaneously and with similar volume.

### Limitations

Our study is limited by being single-center, retrospective, and nonrandomized. As a retrospective cohort study, we find imbalances between our two cohorts with regard to certain demographic factors. Although we attempted to match our cohorts by malignant pathology, we recognize that those undergoing MIDP had a significantly lower incidence of PDA and truly invasive cancers; this is due in part to the practice patterns of the surgeons at our institution, who prefer to use minimally invasive approaches for less aggressive disease. We did not find any statistically significant imbalances in other possible confounders—diabetic status, smoking history, BMI, and so on—between the two groups.

However, we were particularly limited in our ability to include cases of ductal adenocarcinoma in the MIDP group. Patients with invasive cancers do appear to have a higher mean NLR than those without, but this appears to be disproportionately true for patients undergoing open surgery. The converse is true for patients with PNETs, who appear to have a lower mean NLR overall but especially so in those with PNETs undergoing minimally invasive surgery. Although we are confident that surgical approach has an effect on NLR independent of pathology, we opted to omit overall and disease-free survival analyses due to this imbalance in pathology, particularly given the low incidence of PDA in the MIDP group.

In addition, the total sample size of our study was limited by our desire to keep the measurement of postoperative NLR consistent with regard to timing after surgery. However, the primary focus of this study was to investigate the relationship between surgical approach and NLR, establishing benchmarks for further use of NLR in the postoperative setting; further study of the impact of NLR on outcomes may become more fruitful as more minimally invasive surgeries are performed for all kinds of pathologies.

Finally, we focus on distal pancreatectomy and splenectomy, and although we may expect our results to be applicable to all kinds of minimally invasive surgery, this has yet to be proven. There is some evidence that suggests that performing a splenectomy concomitantly with another procedure could blunt the NLR response,^24^ although this should not have a significant impact on the internal validity of our data as all subjects in the study had a splenectomy performed at the time of distal pancreatectomy. In addition, we were unable to compare changes in preoperative NLR, as it is not routine practice for our surgeons to obtain a CBC with differential before surgery; future prospective studies of this topic could illustrate the trend of NLR as a response to open or minimally invasive surgery, respectively.

## Conclusion

The NLR is a validated and easily accessible marker of postoperative inflammation that is lowered by the use of minimally invasive techniques, although NLR may be limited in its ability to predict complications and outcomes after distal pancreatectomy. Further study incorporating more patients with ductal adenocarcinoma and longer follow-up is needed to elucidate the role of NLR as a prognostic marker for postoperative outcomes and overall survival.

## Abbreviations Used

BMI: body mass index
CBC: complete blood count
CRP: C-reactive protein
EBL: estimated blood loss
IOPN: intraductal oncocytic papillary neoplasm
IPMN: intraductal papillary mucinous neoplasm
MCN: mucinous cystic neoplasm
MIDP: minimally invasive distal pancreatectomy
NLR: neutrophil-to-lymphocyte ratio
OR: operating room
PDA: pancreatic ductal adenocarcinoma
PNETs: pancreatic neuroendocrine tumors
POD: postoperative day
POPF: postoperative pancreatic fistulae
WBC: white blood cell count
